# Role of the Synthetic B1 Vitamin Sulbutiamine on Health

**DOI:** 10.1155/2020/9349063

**Published:** 2020-04-20

**Authors:** Bernardo Starling-Soares, Pedro Carrera-Bastos, Lucien Bettendorff

**Affiliations:** ^1^Extreme Sports Nutrition Institute, Belo Horizonte, Minas Gerais, Brazil; ^2^Center for Primary Health Care Research, Lund University/Region Skåne, Skåne University Hospital, Malmö, Sweden; ^3^Laboratory of Neurophysiology, GIGA-Neurosciences, University of Liege, 4000 Liege, Belgium

## Abstract

Sulbutiamine is a thiamine derivative developed in Japan in the mid-60's as a beriberi treatment drug. Since then, different potential applications have been described. For instance, there is some evidence that sulbutiamine can have anti-fatigue, nootropic, and antioxidant effects, which led to its use as a sport supplement (although some authors argue it is actually a masking doping strategy). Moreover, this molecule has been proposed as a possible treatment for some microsporidial infections and even for certain types of cancer. Despite these potential effects, sulbutiamine is still a relatively unknown molecule, which justifies the present review, where we discuss its history and the existing literature on its health applications. We conclude that there is a great potential for sulbutiamine use, well beyond its first described function (to increase thiamine tissue concentration). Indeed, new mechanisms of action have been found, mainly associated with its derivatives. Nevertheless, and although the research on sulbutiamine started 50 years ago, only a limited number of studies were conducted during this time frame. As so, methodological concerns need to be addressed and new studies are necessary, especially randomized controlled trials. Only then will the full potential of this versatile molecule be identified.

## 1. Introduction

**Figure 1:**
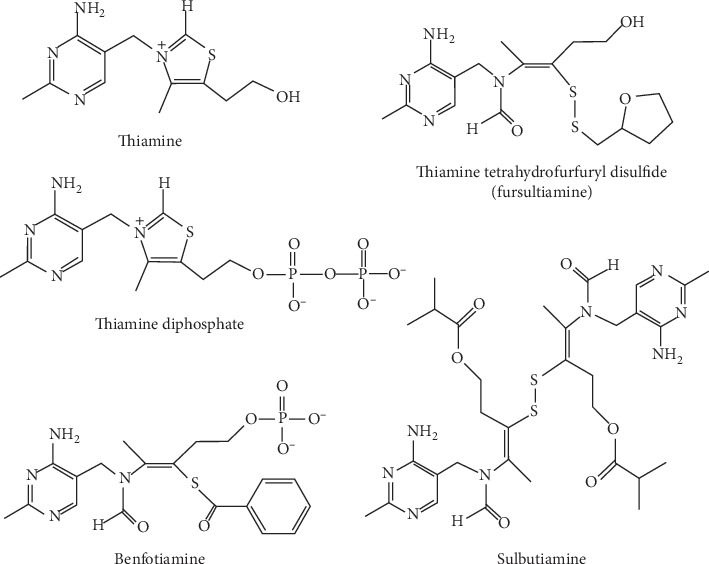
Structural formulas of thiamine, thiamine diphosphate, the thioester benfotiamine, and the disulfide compounds fursultiamine and sulbutiamine.

Sulbutiamine (Figure 1) results from the fusion of two thiamine (Figure 1) molecules linked by a disulfide bridge after the opening of their respective thiazolium rings and esterification of the primary alcohol of each half-molecule by an isobutyryl group [1]. These modifications increase the lipophilic characteristic of this derivative compared to thiamine. Regarding its chemical names, these are: isobutyrylthiamine disulfide, [4-[(4-amino-2-methyl-pyrimidin-5-yl)methyl-formyl -amino] -3-[2-[(4-amino -2-methyl-pyrimidin-5-yl)methyl-formyl-amino]-5-(2-methylpropanoyloxy) pent- 2-en-3-yl]disulfanyl-pent-3-enyl] 2-methylpropanoate), bisibutiamine, and thiamine di(2-methylpropionate) disulfide [1–5]. It is sold under the brand names Arcalion (Laboratoires Servier) and Enerion (Egis Pharmaceuticals) [6, 7] and is commonly mistaken for butyrylthiamine, thiamine propyl disulfide, butyryl thiamine disulfide, and benfotiamine (thiamine precursor with different molecular structures and chemical/biological properties, Figure 1) and even for Vitaberin (actually a berberin-based complement-Ellab Laboratory). Sulbutiamine is also different from thiamine tetrahydrofurfuryl disulfide (Fursultiamine, Figure 1), another pharmacologically active thiamine disulfide compound known to promote voluntary activity through dopaminergic activation in the medial prefrontal cortex [8].

The synthesis of sulbutiamine and the first controlled studies on this molecule were made in Japan in the mid-60s (Tanabe Pharmaceutical-O-isobutyrylthiamine disulfide) [9]. It was mainly developed, along with other lipid soluble thiamine derivatives, for the treatment of beriberi, which was a public health problem in Japan until the 2^nd^ World War [10, 11]. Then, in the 70s, a French pharmaceutical laboratory launched sulbutiamine under the brand name Arcalion labeled as an anti-asthenia drug, notwithstanding the fact that previous work had been focused on intestinal, urological, and cardiac actions of the near cousin of sulbutiamine, BuTDS (O-butyrylthiaminedisulfide) [12–17]. Until that time, there were only assumptions about the intracellular (intraerythrocytary) metabolism of sulbutiamine based on Kawasaki's studies with thiamine propyl disulfide [9, 16, 17]. But since then, it has been established that the uptake of thiamine derivatives by erythrocytes runs in parallel with blood retentivity, and that among the O-acyl thiamine disulfides tested, O-butyrylthiamine was the most efficient. Indeed, after parenteral administration of O-butyrylthiamine, an increase in the half-life of thiamine seems to be the result of its uptake by red blood cells [9]. This observation seems to have been the initial argument for the use of sulbutiamine in thiamine deficiency disorders.

**Figure 2:**
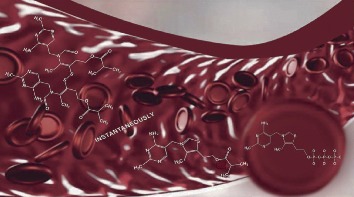
Sulbutiamine transformation in the body. Molecule at left, sulbutiamine; center molecule, O-isobutyryl-thiamine; white inside red blood cell, thiamine triphosphate. ^*∗*^As genuine sulbutiamine was never detected in the blood after oral or intravenous administration, its transformation must be very rapid.

Later, *in vivo* experiments proved that sulbutiamine can be transformed into thiamine derivatives after the reduction of the disulfide bond and regeneration of the closed thiazolium rings, yielding mainly two molecules of isobutyryl-thiamine (Figure 2). Chromatographic behavior of plasma thiamine after the injection of sulbutiamine proved to be exactly the same as for “genuine” thiamine, implying that the isobutyryl groups are removed. This conclusion is more clearly supported by the verified formation of ThMP (thiamine monophosphate), since the isobutyryl group would block the phosphate acceptor group (alcohol group) essential for thiamine phosphate derivatives formation [1]. Another important observation was that sulbutiamine increases the levels of thiamine and thiamine phosphate esters in the brain, thus having the potential to impact asthenia and chronic fatigue [1, 7, 18, 19]. In addition to thiamine, at least four phosphorylated thiamine derivatives have been described in living organisms: thiamine monophosphate (ThMP), thiamine diphosphate (ThDP, Figure 1), thiamine triphosphate (ThTP), and the recently discovered thiamine adenine nucleotide [20, 21]. But the main forms of vitamin B1 in plasma are thiamine and its derivative ThMP, which are transported into cells by specific transporters. ThMP can then be hydrolyzed to thiamine and subsequently pyrophosphorylated to ThDP by thiamine pyrophosphokinase (EC 2.7.6.2). Of interest, ThDP is the most researched thiamine derivative, since earlier studies have clearly demonstrated its role as a cofactor for mitochondrial pyruvate and alpha-ketoglutarate dehydrogenases, as well as for cytosolic transketolase, thereby affecting multiple functional systems [20].

Despite its importance, thiamine transport across cell membranes is a relatively slow process, hence the rationale for the development of lipid soluble thiamine precursors able to freely diffuse into the cells and, especially, across the blood brain barrier. Indeed, because of the role of ThDP as a coenzyme in oxidative metabolism, brain is particularly affected during thiamine deficiency. In this regard, studies conducted in animal models (e.g., rats) have suggested that there are at least two different ThDP pools in the brain: a small pool of high turnover, precursor for ThMP and ThTP, and the larger coenzyme pool with increased half-life (probably because of its binding to apoenzymes) [20]. It is not clear whether these two pools are the result of a compartmentation between different cell types (e.g., neuronal and glial cells) or whether they coexist in the same cell type. As it has been shown that in cultured cells of neuronal origin two ThDP pools (cytosolic and mitochondrial) coexist in the same cell line, the latter is the most probable [20].

ThTP is another thiamine derivative found in many cell types, including bacteria, but it is generally of minor abundance compared to ThDP. Interestingly, administering sulbutiamine to rats leads to increased levels of ThTP in brain tissue. ThTP seems to be preferentially associated with plasma, mitochondrial, and nuclear membranes in neurons, its synthesis previously being related to an “essentially and still not described specific membrane-associated enzyme in the brain” and presenting a role in membrane permeability modulation [1, 22]. Bettendorff and colleagues have shown that mitochondria isolated from rat brain cells synthesize ThTP from ThDP and *P*_i_ using the proton-motive force. This shows that (1) F_0_F_1_–ATPase is a necessary gear of this process and (2) ThTP synthesis is not ATP-dependent but actually P_i_-dependent [22, 23]. ThTP is also synthesized by cytosolic adenylate kinase in skeletal muscle and electric organ and in non-excitable tissues, where this enzyme is particularly abundant [24, 25].

Sulbutiamine can also have neuroprotective effects, mainly because of its thiol content, which can affect antioxidant status. It is well known that the global thiol concentration is important in the regulation of the cellular redox status, since it provides a large antioxidant pool, consisting of (1) thiols bound to proteins, (2) sulfur bound in the disulfide bridges, and (3) free thiol groups, mainly in the form of reduced glutathione (GSH) [26]. Interestingly, several thiol containing compounds, including sulbutiamine, have been shown to upregulate GSH [27], which could prevent oxidative stress in brain cells. However, the mechanisms of action remain controversial.

Sulbutiamine is also a potent inhibitor of triosephosphate isomerase of the protozoan *Encephalitozoon intestinalis* causing microsporidosis, as shown by Garcia-Torres and colleagues [28]. Sulbutiamine specifically inactivates the protozoan enzyme through interaction with cysteine residues, without affecting the human enzyme. This raises the possibility of using sulbutiamine in the treatment of microsporidosis [28]. Since its development, sulbutiamine has been tested for the treatment of several diseases, with mixed results. In retrospect, sulbutiamine was first used in neurochemical studies [29, 30] and, more recently, it has been shown to improve fatigue in patients with multiple sclerosis (MS) who were already being medicated for the disease (e.g., Fingolimod; *β* interferons; Glatiramer acetate; Azatihopurine) [31]. Despite the methodological limitations of this study, this is an important observation and opens the field for sulbutiamine use in various conditions, particularly in those characterized by an impaired intracellular and central nervous system thiamine metabolism [31, 32]. Overall, sulbutiamine appears to have various potential effects, such as improvement of asthenic syndromes, modulation of the wakefulness state (in extreme high doses), enhancement of long-term memory formation, chronic postinfectious fatigue amelioration, anti-ischemic effect, psycho-behavioral inhibition in major depression, modulatory effect on glutaminergic and dopaminergic cortical transmission, improvement of diabetic polyneuropathy and psychogenic erectile dysfunction parameters, reduction of amnesic effects of drug-induced schizophrenia, modulation of Alzheimer's disease, and treatment for microsporidosis caused by the intracellular parasite *Encephalitozoon intestinalis* (Supplementary Table S1) [3, 6, 7, 19, 28–30, 33–39].

## 2. Studies Included in This Review

The purpose of this review is to thoroughly describe sulbutiamine and its health applications, and to give directions for future research. As such, a web-based literature search was performed to identify all papers until January 2020 citing the following search terms: *Sulbutiamine or Sulbuthiamine; Isobutyrylthiamine Disulfide; Arcalion; Enerion; Bisibultiamine or Bisibulthiamine;* [4-[(4-amino-2-methyl-pyrimidin-5-yl) methyl-formyl-amino]-3-[2-[(4-amino-2-methyl-pyrimidin-5-yl)methyl-formyl-amino]-5-(2-methylpropanoyloxy)pent-2-en-3-yl]disulfanyl-pent-3-enyl]2-methylpropanoate; bis-(isobutyryloxy-2-ethyl)-1-N-(amino-4-methyl-2-pyrimidyl-5) methyl formamido-2-propene-1-yl disulfide. The databases used were Pubmed/MEDLINE and ScienceDirect. References of the eligible articles were then verified manually seeking potential additional articles that could have been missed by the electronic search. A total of 85 papers/book chapters/posters were found, but 67 of these were classified as “not related to the health and medical uses of sulbutiamine.” As a result, only 20 citations were included in the results of this review (Supplementary Table S1), although several papers related to sulbutiamine were presented here.

### 2.1. Sulbutiamine and Thiamine Phosphate Metabolism

After chronic intraperitoneal injection of sulbutiamine in rats (52 mg/kg for 14 days), ThDP was found to be the most abundant thiamine compound in the renal cortex, the medulla oblongata (including the pons), the cortex (telencephalon), the cerebellum, and the hippocampus [1]. In brain extracts, its proportion ranged from 55.6% of total thiamine in the cytosolic fraction to 90.4% in the homogenate [20]. Interestingly, a more recent study from Sambon and colleagues reported that the maximum ThDP content in cultured neuroblastoma cell line grown in a thiamine-restricted medium is achieved more rapidly with the addition of sulbutiamine, when compared to thiamine and benfotiamine [40]. As for ThTP, it exists in small amounts (0.1–1% of total thiamine) in animal tissues [1]. Nevertheless, a remarkable study on the metabolism of thiamine phosphate conducted by Bettendorff in 1994 shed new light on this issue. He observed that, after incubation of cultured neuroblastoma cells with radioactively labeled sulbutiamine, the radioactivity increased more rapidly in ThTP and ThMP than in their precursor (ThDP), suggesting the existence of two ThDP intracellular pools: one high turnover pool precursor for ThMP and ThTP synthesis and one low turnover pool of ThDP associated with apoenzymes. Furthermore, sulbutiamine was about 10 times more effective in increasing intracellular thiamine concentrations than thiamine. However, the increases in intracellular ThDP and ThTP concentrations were barely significant after 4 h, suggesting a tight regulation of ThDP synthesis. These data show that once sulbutiamine enters the cells it is hydrolyzed and quickly reduced to thiamine, which may then be incorporated in thiamine phosphate derivatives. Indeed, a 30% increase in ThTP levels was seen in the first minutes and it reached a specific radioactivity close to that of thiamine after a 4 h exposure, showing that an equilibrium is achieved [18]. Following this same line, Bettendorff's group presented in the same year a study using young Female Wistar rat brain fractions to show that a relatively rapid ThTP was observed when the animals received sulbutiamine and that this synthesis was linked to synaptoneurosomes and mitochondria [20]. However, the complete mechanism of ThTP synthesis has been a puzzle that only now starts to be solved [22, 25].

### 2.2. Neurochemical Actions of Sulbutiamine

Using very high doses (300 mg/kg/day) of sulbutiamine in Rhesus Monkeys (*Macaca mulatta*), Balzamo & Vuillon-Cacciuttolo have observed an increase in the vigilance pattern [29]. Nevertheless, it is not entirely clear whether these effects were due to increased brain thiamine phosphate levels or to another compound [20]. Whatever the exact mechanism, however, other studies have confirmed the neurological effects of sulbutiamine. For instance, when applied intraperitoneally in mice, at doses close to human therapeutic doses, sulbutiamine had modulatory effects on glutamatergic and dopaminergic central transmission changing the turnover of both receptors and neurotransmitters. It should, however, be mentioned that these effects appear to depend on whether it is applied chronically or acutely. As an example, after administering this molecule to rats for 5 days, there was a significant increase in the density of dopamine D1 receptor binding sites in prefrontal and anterior cingulate cortex (+26% and +34%, respectively), as well as a significant decrease in the density of kainate binding in the cingulate cortex, the nucleus accumbens (−36% and −28%, respectively), striatum, and hippocampus. On the other hand, the acute administration of sulbutiamine decreased the density of kainate binding sites in prefrontal and cingulate cortex, reduced 3,4-dihydroxyphenylacetic acid (DOPAC) levels (−30%) in the prefrontal cortex, and decreased dopamine (DA) and DOPAC levels (−34% and −26%) in the cingulate cortex [36].

### 2.3. Memory and Sulbutiamine

Chronic administration of moderate to high doses of sulbutiamine to BALB/*c* mice increased sodium-dependent high affinity choline uptake by 10% in hippocampal neurons and improved performance in a partial acquisition task but not the capacity of acquisition in a single test (learning rate) [30]. Since the hippocampal cholinergic system is associated with long-term memory formation, these results prove that moderate to high doses of sulbutiamine are capable of enhancing long-term memory formation in mice. Again, it is not yet known whether these properties are mediated through the action of thiamine phosphate derivatives or are the result of thiamine disulfide specific reactions [20]. Independently of the exact mechanism, when tested for its capacity to interfere with the detrimental effects of dizocilpine-induced amnesia, on acquisition, consolidation, and memory retention of rodents, the chronic use of sulbutiamine showed memory improvement in an object recognition task and reduced the disruption of working memory and the deficit of acquisition of information stored in episodic memory [38]. And in one of the few human trials ever conducted that evaluated memory parameters, sulbutiamine (400–600 mg/day), in association with a cholinesterase inhibitor treatment (donepezil 5–10 mg/day), improved episodic memory, attention, and daily activities, when compared with donepezil alone. But when comparing the effects of sulbutiamine and donepezil separately, a similar improvement between groups was seen only in the test evaluating attention. This was a multicentric, randomized, and double-blind trial engaging early-stage Alzheimer disease patients [39].

### 2.4. Fatigue and Sulbutiamine

Since the beginning of its commercialization, sulbutiamine has been considered a nootropic molecule. Following Micheau's observation that sulbutiamine exhibits a cholinergic hippocampal modulation and that this could be linked to partial memory improvement in mice, this drug started to be prescribed to treat fatigue conditions [30, 31]. But Tiev and colleagues showed, in 1999, in a randomized, double-blind, parallel group, placebo-controlled study, that although an improvement was observed on the 7th day, doses of 600 mg/day did not have a persistent effect at the end of 28 days, in women suffering from post-infection asthenia [19]. This is in contrast to an Indian prospective uncontrolled, non-randomized, commercial, observational study, where 52% of the patients presented complete resolution of infection-induced fatigue when sulbutiamine was used in combination with an anti-infective treatment [33]. Although the authors suggested that increases in acetylcholine transmission and consequently the level of activation of the ascending reticular formation level (potentially produced by sulbutiamine or some derivative) may be related to the pathogenesis of asthenia and the improvement seen, there is a need to evaluate to what extent asthenias related to acute infections may be naturally improved with the amelioration of the disease. Notwithstanding these conflicting results, a recent retrospective observational study, conducted by Sevim et al., concluded that sulbutiamine use was associated with a reduction in the total score of Fatigue Impact Scale (FIS) and in all other three subscales assessing physical, cognitive, and psychosocial functioning in multiple sclerosis patients who were on some kind of disease modifying treatment. Nevertheless, in patients who were not on any treatment, 400 mg/day of sulbutiamine did not show any improvement [31].

### 2.5. Depression and Sulbutiamine

Although no antidepressant effect of sulbutiamine was reported in patients with major depressive episodes, a multicentric, randomized, double-blind, placebo-controlled trial conducted by Loo et al. has shown that the administration of 600 mg/day of sulbutiamine for 8 weeks significantly decreased social inhibition associated with the depressive event [35].

### 2.6. Diabetes Polyneuropathy and Sulbutiamine

Kiew and colleagues using 400 mg/day of sulbutiamine for 42 days in an open-label randomized controlled study showed improvement in nerve signal parameters in type 2 diabetes patients suffering from polyneuropathies. However, there was no significant change in metabolic biomarkers, such as blood glucose and HbA1 [6]. Moreover, this study had several methodological limitations. Therefore, further research is needed before a definitive conclusion can be reached.

### 2.7. Psychogenic Erectile Dysfunction and Sulbutiamine

In a Hungarian study, Dmitriev and colleagues evaluating the efficacy of sulbutiamine treatment for 30 days in patients with psychogenic erectile dysfunction showed improvement in specific parameters of this disorder. The International Index of Erectile Function increased from 17.5 to 25.8, a 50% improvement in patients suffering from cavernous arterial dysfunction. Moreover, 36% of the patients experienced normalization of erectile function [37]. This study was not a controlled trial and, to our knowledge, it has not been replicated, and as such these results should be viewed with caution.

### 2.8. Deprivation Models and Sulbutiamine

Using the premises that “serum deprivation procedure has been used in various experimental models to induce neuronal death” and “serum withdrawal reduces the levels of neurotrophins and antioxidants in the extracellular medium which eventually leads cells to “starve” from these neurotrophic factors,” Kang and colleagues presented the beneficial actions of sulbutiamine in serum-deprived transformed retinal ganglion cells [27]. These effects were mainly due to enhancement of the cellular antioxidant and anti-apoptotic capacities by sulbutiamine and/or its derivatives, emphasizing its neuroprotective properties. Although 50 *μ*M was the most effective dose when cell viability in a serum-deprived context was measured, lower doses (1 and 10 *μ*M) of sulbutiamine showed very similar and promising results [27]. In 2011, this same Korean group using an ischemic model (oxygen-glucose deprived serum) in rat hippocampal CA1 pyramidal neurons showed increased neuronal viability, intrinsic membrane input resistance, and excitatory synaptic transmission in a dose dependent manner. 50 *μ*M was again the most effective dose [41]. Working with the same *in vitro* model (transformed retinal ganglion cells-RGC-5 cells), Majid (one of the researchers of the two previous studies) and colleagues showed a dose dependent increase in cell viability (0%, 18%, and 28% with, respectively, 10, 50, and 100 *μ*M sulbutiamine), less apoptosis, less caspase-3 activity, and a decrease in ROS levels for the 100 *μ*M dose. Sulbutiamine at 100 *μ*M also produced increases in catalase, GSH, GST (glutatione-s-transferase), Nrf-2 (nuclear factor erythroid 2-related factor 2), and HO-1 (Heme oxygenase-1) levels in serum-deprived medium. Of notice, when an oxidant medium was created using glutamate/buthionine sulfoximine no difference was seen in the majority of the parameters evaluated. In this later model, N-acetylcysteine (which is a precursor of GSH) showed better results and thus may be a complementary strategy to sulbutiamine in conditions associated with elevated oxidative stress (e.g., ischemia, diabetes) [42]. Benfotiamine is another synthetic derivative of thiamine that has been pointed as a potent stimulant of the antioxidant system. In an elegant study, Tapias and colleagues showed that benfotiamine and its main derivatives, but not thiamine, increased the expression of Nrf2/antioxidant response element (ARE)-dependent genes in mouse brain. This suggests that the disulfide bridge is not necessarily essential for the antioxidant action of sulbutiamine [43]. A recent study using a mouse neuroblastoma cell line (Neuro2a) grown in thiamine-restricted medium for 7 days and evaluating cell survival rate using the MTT method presented that the protective effects of benfotiamine and sulbutiamine are directly related to thiamine levels when cells were stressed by exposure to paraquat and *β*-amyloid peptide. This suggests these compounds act through a coenzyme-independent and indirect ROS scavenging mechanism [44]. Multiple positive interactions and effects between thiamine and derivatives of its different analogs appear to be possible when distinct deprivation models are tested. Therefore, the design of new studies and the discovery of theses derivatives will allow us a better understanding through which mechanisms and pathways each related thiamine molecule can act.

### 2.9. Microsporidiosis and Sulbutiamine

In a very different field, Garcia-Torres and colleagues discovered that low concentrations of sulbutiamine can specifically inhibit *Encephalitozoon intestinalis* triosephosphate isomerase and as such have classified this molecule as “a promising drug in the treatment of brain microsporidiosis (encephalitis),” one of the complications of this parasite that lacks mitochondria and oxidative phosphorylation. This discovery could have important consequences, since the prevalence of this type of infection can reach 60% in developing countries, Mexico being the country with the highest rate [28]. However, and although these researchers mention the ability of sulbutiamine to cross the blood-brain barrier, this molecule has never been identified in animal brain in its natural chemical structure after oral or even parenteral administration. Thus, it seems that other ways of administration will be needed to direct sulbutiamine to the brain. Nevertheless, other explanations are possible, namely, (1) after interacting with *Encephalitozoon intestinalis* in the intestinal tract, sulbutiamine may decrease the infectious rate by some unknown action; (2) some presently unidentified metabolites of sulbutiamine reaching the brain could interact with the cysteines of the enzyme thus inhibiting its activity.

### 2.10. Anticancer Potential of Sulbutiamine

Proliferation of cancer cells is often related to high activity of the key mitochondrial metabolism enzyme pyruvate dehydrogenase kinase (PDK), the so-called Warburg effect. Thiamine has been cited as a potential inhibitor of tumor proliferation *in vivo* [44] presumably by inhibiting PDK function and consequently enhancing pyruvate dehydrogenase (PDH) and mitochondrial function. Therefore, it may be limited by transporter saturation and restricted accumulation of thiamine within tumor cells. Aiming to circumvent this, the lipophilic thiamine analogs sulbutiamine and benfotiamine were tested in cultured human cancer cell lines and its cellular proliferation was determined using a crystal violet assay. The sensitivity of various tumor cell lines (HCT 116 (colorectal carcinoma), U-87 MG (glioblastoma), MDA-MB-231 (metastatic breast adenocarcinoma)) determined by crystal violet assay showed a decreased proliferation for thiamine, sulbutiamine, and benfotiamine after 5 days of treatment. The main difference was the concentration necessary for the effect (IC_50_), being 30–50-fold lower for sulbutiamine and benfotiamine than for thiamine for all cancer cell lines tested. When enzymatic specific PDH activity tests were done, no effect for thiamine, TMP, sulbutiamine, or benfotiamine was observed, and only ThDP was capable of decreasing the PDH inhibition made by PDK isoforms. When *in vivo* tumor growth-induced mice (subcutaneous xenograft model) were treated every second day with pharmacological doses (250 mg/kg) of sulbutiamine or benfotiamine, both compounds increased the ThDP content, but only benfotiamine reduced the tumor volume and weight. Neither sulbutiamine nor benfotiamine was detected in the tumors, suggesting that the apoptotic response observed following benfotiamine treatment is different from sulbutiamine, probably by its specific derivatives and independent of the PDH-PDK axis [45]. Although this novel and preliminary study of Jonus and colleagues presents sulbutiamine as an anticancer therapy *in vitro* but not *in vivo,* two points cited by the authors have to be considered before excluding its *in vivo* potential:

1. Changes to PDH phosphorylation may have been impacted by the terminal end point used for analysis of tumor tissue and not indicative of the initial response to treatment that would be more complementary to *in vitro* findings
2. Alternatively, the increase in TPP achieved by *in vivo* dosing paradigm may not have been substantial enough to sufficiently inhibit PDK

Therefore, new studies need to be done using sulbutiamine in cancer models to address the potential anticancer effect of sulbutiamine.

### 2.11. Antidoping and Sulbutiamine

The sulbutiamine mass spectrometry peak is cited as one of the “peaks identified as a compound with a possible anabolic, or otherwise stimulating or endurance enhancing effect” by Peters and colleagues [4]. In this study aimed at developing a novel technique to identify anabolic steroids, herbal mixtures and sport supplements were used as samples. Later, in the same year, Sobolevsky & Rodchenkov, researchers of the Moscow Antidoping Center, observed that 2% of 5,151 antidoping urine samples contained sulbutiamine and alerted that “sulbutiamine could hamper the detection of boldenone metabolites due to the coelution when a single quadrupole GC-MS instrument is used for the screening analysis” [5]. However, as an analytic study this paper gives no information as to the route of administration and the dose used so as to find a significant concentration in the urine (>500 ng/ml or >0.7 *μ*M). Previous attempts in rodents were unsuccessful and it is not clear whether this is due to more sensitive analytical tools or the fact that it was done in humans and not in rodents. But this paper may be the document proof of the existence of sulbutiamine in body fluids.

## 3. Side Effects

Balzamo and Vuillon-Cacciuttolo reported hypersalivation in 2 out of 6 rhesus monkeys after administration of high doses of sulbutiamine (300 mg/kg/day). Although these high doses affected the sleep pattern, they did not alter the motor ability of the animals [29]. Shah reported side effects occurring in 0.6% of the patients using sulbutiamine. In a total of 1,772 patients, 5 presented nausea, 1 headache, 1 insomnia, 1 diarrhea, 1 tremor, and 1 drowsiness [33]. Sevim and colleagues reported no “serious” adverse effect in 26 relapsing-remitting multiple sclerosis patients using a single dose of 400 mg of sulbutiamine [31]. In accordance, in a sample of 300 patients included in the study (approximately 100–400 mg sulbutiamine; 100–600 mg sulbutiamine; 100–placebo), Tiev and colleagues reported that only 15 patients using sulbutiamine presented side effects (headache, agitation, palpitations, diarrhea, cough, sinusitis, cystitis, bronchitis, osteoarthritic and low back pain, palpitations, asthma, abdominal pain, and nephrotic colic). However, these side effects may not be related to sulbutiamine as in the placebo group 12 patients presented similar effects [19]. In another study, Ollat and colleagues showed that 3 patients out of 40 (8%) using sulbutiamine in a standalone therapy had headache/insomnia as a side effect [39].

## 4. Conclusions

In recent years, there has been an increase in the number of studies on the health applications of sulbutiamine. Experimental evidence, observational studies, and clinical trials suggest that sulbutiamine has various actions in human physiology through its ability to provide thiamine to less accessible tissues (such as the brain), by increasing antioxidant capacity and by modulation of protein action [7, 41]. However, caution is required in evaluating some of these results (e.g., post-infection fatigue will be necessarily ameliorated in the first month even in patients not using sulbutiamine, and as pointed by the authors “relief from asthenic symptoms cannot be attributed exclusively to sulbutiamine”) [33, 46, 47]. There is also evidence that sulbutiamine has, to some degree, a neuromodulator capacity (probably through some of its metabolites). Nevertheless, various questions and concerns must be answered by controlled studies before the effectiveness and clinical relevance of sulbutiamine can be attested.

The dosage and absorption rates seem to be another important question to address in future studies, as seen in Balzamo's study, where sulbutiamine at approximately 10 times the usual dose interfered with the sleep/wake cycle. However, low doses (1–10 *μ*M) used in *in vitro* studies showed general antioxidant effects, although 50 *μ*M could have additional effect in neurons. One explanation for this could be that ThTP content generated by sulbutiamine could play a role in relation to membrane permeability modulation [1].

It is also important to remark that sulbutiamine seems to have the capacity to interfere with the action of central proteins, as in the case of caspase-3, bearing in mind that “the primary caspases involved in the apoptotic degeneration of RGC-5 after axotomy have been caspase-3 and caspase-9” [42]. Even more interesting, sulbutiamine, as presented by Garcia-Torres and colleagues, may inactivate microsporidial triosephosphate isomerase, putting this molecule in a class of drugs and supplements with a broad spectrum of action [28]. And now it will probably gain attention in oncologic research as the most recent publication presented an anticancer effect *in vitro* [45].

Because of the great potential of this molecule, initially designed for the treatment of beriberi, gaps in the literature should not intimidate but challenge researchers to design and conduct high quality research. This review intends to present and discuss most of information available on sulbutiamine and possibly serve as a starting point for future research. Specifically, randomized controlled trials are necessary to investigate the health potential of this molecule. Presently, the safest approach seems to lie in evaluating patients individually and carefully considering the possibility of using sulbutiamine in adequate doses for the clinical conditions already listed here. Also, it would be of interest to compare the effects of sulbutiamine with those of thiamine and benfotiamine (which is not a disulfide, but a thioester) both *in vitro* and *in vivo*. This would help answer the question whether sulbutiamine mainly acts via thiamine/thiamine phosphate derivatives or whether it has more specific pharmacological effects. Efforts should be undertaken to investigate whether, after administration of sulbutiamine, this compound or its metabolite thiamine disulfide can be found in the brain using modern mass spectrometry-based analytical methods.

## Supplementary Materials

Supplementary Materials Description of sulbutiamine studies and its health applications.
